# The potassium puzzle: exploring the intriguing connection to albuminuria

**DOI:** 10.3389/fnut.2024.1375010

**Published:** 2024-05-27

**Authors:** Weihua Yuan, Tiancheng Wang, Wei Yue

**Affiliations:** ^1^School of Acupuncture-Moxibustion and Tuina, Anhui University of Chinese Medicine, Hefei, China; ^2^School of Integrated Traditional and Western Medicine, Anhui University of Chinese Medicine, Hefei, China; ^3^Yancheng TCM Hospital Affiliated to Nanjing University of Chinese Medicine, Yancheng, China

**Keywords:** albuminuria, potassium, dietary element intake, NHANES, cross-sectional study

## Abstract

**Background:**

Studies have revealed a relationship between dietary potassium intake and albuminuria, despite the fact that the human body needs a lot of potassium. Our study concentrated on the link between dietary potassium intake and albuminuria.

**Methods:**

This study used subgroup analysis and weighted multivariate regression analysis. Data from the National Health and Nutrition Examination Survey (NHANES) were examined to determine the urinary albumin-to-creatinine ratio (ACR) and participant age (20 years or older). ACR >30 mg/g was the threshold for albuminuria.

**Results:**

7,564 individuals in all were included in the study. The link between the two was significant in both our original model (OR = 0.99; 95% CI, 0.98–0.99, *p* < 0.0001) and the minimum adjusted model (OR = 0.99; 95% CI, 0.98–0.99, *p* < 0.0001). A fully adjusted model did not change the significance of the negative correlation between potassium consumption and albuminuria (OR = 0.99; 95% CI, 0.98–1.00, *p* = 0.0005), indicating that each unit increase in potassium intake was related with a 1% decrease in the chance of developing albuminuria. The negative correlation between potassium intake and albuminuria was not significantly influenced by sex, age, BMI, hypertension, diabetes, or smoking, according to interaction tests (*p* for interaction >0.05).

**Conclusion:**

Reduced risk of albuminuria was linked to higher dietary potassium intake. The particular mechanism linking the two still has to be explained by several inventive and prospective studies.

## 1 Introduction

Albuminuria is the amount of albumin in the urine that exceeds the normal range and is often an important indicator of kidney disease. The kidney is one of the important organs in the human body, which is mainly responsible for excreting waste and regulating water, electrolyte and acid–base balance in the body. Albumin is an important plasma protein synthesized by the liver, reabsorbed by the renal tubules after glomerular filtration, and finally excreted from the body. When the glomerular filtration membrane is damaged or the renal tubule reabsorption function is reduced, it causes albumin to leak out of the urine, forming albuminuria. Potassium is one of the essential minerals in the human body and plays an important role in maintaining the normal function of the heart, muscles and nervous system. The kidney is one of the main organs regulating the balance of potassium in the human body, and maintains the stability of potassium in the body through excretion and reabsorption. When kidney function is impaired, potassium metabolism is disrupted, which in turn affects the normal function of the heart, muscles and nervous system. Recent studies have found that there is a certain relationship between potassium and albuminuria (1). Some studies have found that moderate potassium intake can reduce the risk of albuminuria, possibly because potassium promotes the reabsorption of albumin by the renal tubules, reducing albumin leakage from the urine. In addition, other studies have found that excessive potassium intake may increase the risk of albuminuria, possibly because excess potassium can cause a heavier burden on the kidneys, which in turn affects the function of the glomerular filtration membrane. In summary, there is a relationship between potassium and albuminuria, and moderate potassium intake may reduce the risk of albuminuria, but excessive potassium intake may increase the risk of albuminuria. Therefore, it is recommended that people according to their health conditions and eating habits, moderate intake of potassium. If you have kidney disease or other health problems, dietary adjustments and treatment should be made under the guidance of your doctor.

Albuminuria independently predicts cardiovascular disease (2) and chronic kidney disease (3). The ratio of urine albumin to creatinine (ACR), which is used to determine whether or not a person has albuminuria (increased excretion of urinary albumin), must be greater than 30 mg/g (4). Albuminuria can be detected using a variety of techniques, such as standard urine testing, protein qualitative analysis, urine microalbumin detection, etc. ACR detection is currently a relatively quick and accurate process, nevertheless (5). Multiple studies have found that albuminuria has become a serious public health problem, with The median prevalence of ACR ≥30 mg/g across cohorts was 32.1% in diabetes and 21.8% in hypertension (6). Therefore, research into albuminuria is crucial.

Potassium is a chemical element, located in the fourth period of the periodic table (7). Potassium is very active chemically and is a white metal with a soft texture, low boiling point, and melting point, and lower density than water (8). Potassium intake is very important to the body (9), serving as an electrolyte, balancing body fluids, and controlling muscles (10). A lack of potassium at the same time can lead to irregular heartbeats (11), insomnia (9), muscle cramps (12), and fatigue (13). In community-dwelling Japanese people, Kabasawa et al. discovered that dietary potassium intake was linked to higher albuminuria (14). According to Sharma et al., higher sodium and potassium intake is associated with a lower incidence of CKD in U.S. adults. These results should be confirmed by longitudinal studies and specially designed clinical trials to examine the effects of dietary sodium and potassium intake on kidney disease and its progression (15). It is possible to include dietary potassium consumption as an exposure variable because the association between potassium intake and albuminuria has been studied in Japan.

Dietary potassium intake and albuminuria have a complex relationship. CKD progression was found to be substantially faster in hyperkaluric people than in hypokaluric people in a study of patients with CKD stages 2–4 and the association between urine potassium and CKD progression and all-cause mortality (16). Another study discovered that the glomerular filtration rate was unrelated to the substantial association between each 1,000 mg increase in daily dietary potassium intake and a 29% reduction in the probability of developing proteinuria (17). Higher levels of the dietary protein-to-potassium ratio (NEAP) were linked to albuminuria and inversely connected with potassium consumption, according to research by Kabasawa et al. (18). However, we discovered contradictory findings from Kabasawa et al. twice about the link between albuminuria and potassium consumption.

So, we looked into the connection between albuminuria and potassium consumption. Additionally, it was proposed that a higher incidence of albuminuria was linked to a decreased potassium intake.

## 2 Materials and methods

### 2.1 Data sources and participants

The NHANES study evaluates children’s and adults’ nutritional and health status in the US. Children and adults were excluded from this study at the age of 20, and the evaluation study comprised data from questionnaire interviews, tests, and laboratory measurements (19). The board of the NCHS Research Ethics Review has approved the NHANES program of the National Center for Health Statistics. Legal guardians of survey participants under the age of 16 have additionally given written informed consent.

In this study, two cycles of NHANES, 2017–2018 and 2019–2020, were selected. In this study, the 2017–2018 cycle and the 2019–2020 cycle are selected, which are the two most recent cycles with new data. At the same time, this study selects two cycles of data volume to meet the requirements of data statistics. Not only these two cycles included complete dietary potassium intake and albuminuria data, but also the National Center for Health Statistics (NCHS) had merged data for these two cycles, and the data were reliable.

**Figure 1:**
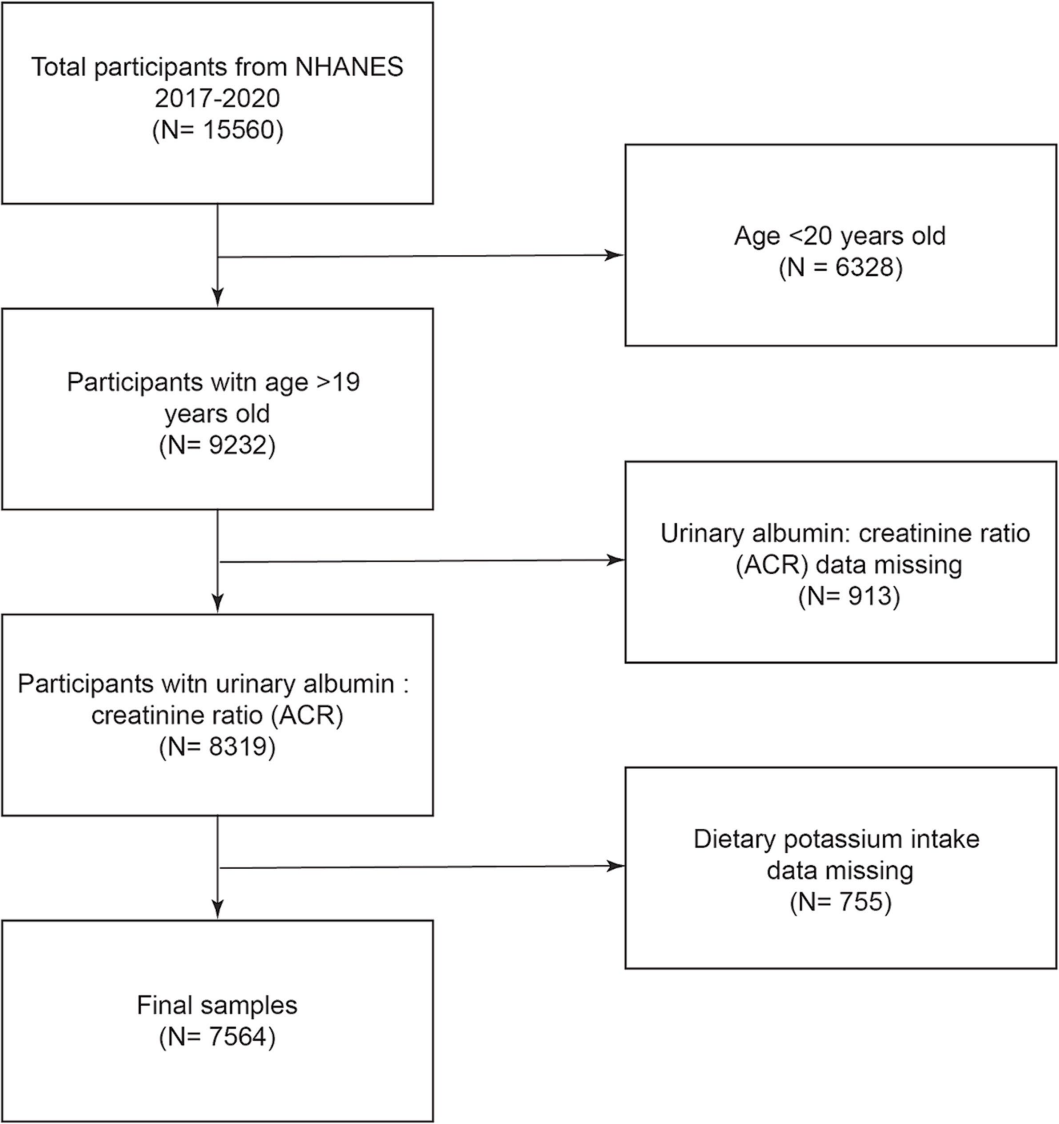
NHANES participant selection flowchart for 2017–2020.

A flowchart of the research population selection is shown in Figure 1. According to the inclusion and exclusion criteria, this study excluded 6,328 people under the age of 20 from the 15,560 participants, excluded 913 people with missing creatinine ratio data, excluded 755 people with missing dietary potassium intake data, and finally included 7,564 people in the study.

### 2.2 Definition of dietary potassium intake and definition of albuminuria

It was selected from the total nutrient intake on day 1 from diet interview data. Interviews were conducted in a fixed environment set up by NHANE, and subjects were provided with a set of test guidelines for reporting food intake. Dietary potassium intake in this study was set as the exposure variable in milligrams.

In this study, the variable URDACT was selected from laboratory data. The urine albumin/creatinine ratio in mg/g (URDACT) was calculated by dividing the urine albumin by the urine creatinine value and multiplying it by 100 (rounded to 2 decimal places). The simultaneous measurement of human urinary albumin is a solid-phase fluorescent immunoassay. Albuminuria was defined as URDACT >30 mg/g (20). Albuminuria was set as an outcome variable in this study.

### 2.3 Covariates

In this investigation, factors that could influence the inverse relationship between dietary potassium intake and albuminuria were included. Covariates include age, poverty-income ratio, total energy intake, total protein intake, dietary carbohydrate intake, dietary sugar intake, dietary total fat intake, dietary cholesterol, dietary calcium intake, caffeine intake, DBP, BMI, fasting blood glucose (21), serum creatinine (22), globulin, Triglycerides (23), uric acid (24), gender, race, education, marital status, drinking level, Hypertensive (25), exercise level, smoking level, etc. BMI, systolic and diastolic blood pressure are important indicators in health assessment, and they were used to analyze the health status of participants in the NHANES study. First of all, BMI (Body Mass Index) is a value obtained by dividing weight (kilograms) by the square of height (meters), used to assess the weight status of an individual. In NHANES, BMI is used to determine whether adults and children are in a healthy weight range, or whether they are underweight, overweight or obese. Second, Systolic Blood Pressure (SBP) refers to the highest pressure in the arteries when the heart contracts, often referred to as “high pressure.” When the heart contracts to pump blood into the arteries, the pressure inside the arteries rises and the blood pressure measured is called systolic. In NHANES, a measurement of systolic blood pressure is used to assess the risk of cardiovascular disease, and it is generally believed that a systolic blood pressure greater than or equal to 140 mmHg in adults may indicate an increased risk of hypertension. Finally, Diastolic Blood Pressure (DBP) is the lowest pressure in the arteries when the heart diastates, also known as “low pressure.” When the heart diastates and blood flows back to the heart, the pressure in the arteries decreases, and the blood pressure measured at this time is called diastolic pressure. In NHANES, diastolic blood pressure measurements are also used to assess the risk of cardiovascular disease, and it is generally believed that diastolic blood pressure greater than or equal to 90 mmHg in adults may indicate an increased risk of hypertension. These indicators are important for understanding the health status of the U.S. population, and they can help public health policymakers develop targeted health promotion programs and interventions. Through these data, we can monitor the prevalence trend of chronic diseases such as hypertension and obesity in the population, so as to provide a scientific basis for the prevention and control of these diseases. The income-to-poverty ratio in NHANES is a measure used to assess participants’ economic status and standard of living. Data on the income-to-poverty ratio is one of the important socioeconomic variables in the NHANES survey. These data are often used to analyze the relationship between health outcomes and economic status, as economic level has a direct impact on the health status of individuals and families. Alcohol consumption levels were assessed as to whether subjects had ever drank 4 to 5 drinks or more per day during their life, 4 drinks or more for women and 5 drinks or more for men. The exercise status questionnaire was set as follows: Does the profession require exerting high levels of effort for at least 10 min at a time, such as lifting or carrying heavy objects continuously, digging, or building work? Selecting smoking status, using a questionnaire, have you ever smoked at least 100 cigarettes in your life, including other tobacco use? 9th through 11th grade (including 12th grade without a diploma). Systolic blood pressure and diastolic blood pressure are, respectively, selected from the average value of three consecutive blood pressure measurement data.

### 2.4 Statistic analysis

Statistical studies were done using the R language (version 4.1.0) and EmpowerStats (version: 4.0). The baseline table of the research population was counted by dietary potassium groups, continuous variables were represented by means and positive and negative standard deviations, and weighted linear regression models were used to describe them.

Different models of the relationship between dietary potassium intake and albuminuria were tested using multivariate logistic regression. Race, age, and gender factors were adjusted in Model 2 but not in Model 1, Model 3 adjusted covariates poverty income ratio, total dietary energy, protein, dietary carbohydrate, sugar, fat, dietary cholesterol intake, dietary calcium intake, caffeine intake, DBP, BMI, fasting blood glucose, serum creatinine, globulin, Triglycerides, uric acid, gender, race, education, marital status, drinking level, hypertensive, exercise level, smoking level. In addition, stratification criteria for the subgroup analysis of the association between dietary potassium intake and albumin birds include gender, educational attainment, smoking status, diabetes, and hypertension. For continuous variables, the median was used as a replacement, and for categorical variables, the mode. Statistical significance is defined as *p* < 0.05.

## 3 Results

### 3.1 Baseline characteristic

According to the inclusion and exclusion criteria, there were 7,564 participants over the age of 20 in this study, and the average age of the participants was 50.62 ± 17.33 years old. The subjects in this study were 48.69% male, 51.31% female, 11.63% Mexican American, 26.72% non-Hispanic black, 35.75% non-Hispanic white, 10.23% other Hispanic, and 15.67% other races. The average dietary potassium intake was 2519.81 ± 1271.07 mg.

Table 1 presents the baseline characteristics of the dietary potassium intake tertile pairs. Among the tertile of dietary potassium intake, poverty-income ratio, total energy, marital status, total protein, gender, carbohydrates, education level, total sugar, total fat, dietary cholesterol, dietary calcium, race, caffeine, DBP, BMI, uric acid, fasting blood glucose, serum creatinine, globulin, Triglycerides, drinking level, hypertensive, exercise level, smoking level, and albuminuria were all statistically significant (*p* < 0.05). At the same time, 13.59% of subjects were classified as having increased urinary albumin excretion, which decreased with increasing dietary potassium intake. Overall, the third quartile of dietary potassium intake and the part with higher intake are more likely to have a low poverty-income ratio, high dietary total energy intake, high protein intake, high carbohydrate intake, high total sugar intake, high total fat intake, high cholesterol intake, high calcium intake, high caffeine intake, low DBP, low BMI, high serum creatinine, low globulin, high Triglycerides, high uric acid, male, non-Hispanic white, Some college or AA degree, Married/Living with Partner, non-drinking, non-hypertensive, non-participating in strenuous exercise, non-smoking, non-albuminuric subjects (see Table 2).

**Table 1:** Baseline characteristics of dietary potassium intake by quantile for the study population.

Dietary potassium intake	Tertile 1 (*n* = 2,515)	Tertile 2 (*n* = 2,526)	Tertile 3 (*n* = 2,523)	*p*-value
Age (years)	49.98 ± 17.61	51.02 ± 17.42	50.85 ± 16.93	0.110
Income of poverty ratio	2.32 ± 1.56	2.73 ± 1.62	2.82 ± 1.66	<0.001
Energy (kcal)	1411.49 ± 565.16	2065.69 ± 668.15	2905.50 ± 1121.18	<0.001
Protein (gm)	49.03 ± 20.98	76.45 ± 26.43	112.38 ± 48.01	<0.001
Carbohydrate (gm)	167.38 ± 80.16	237.40 ± 92.11	332.47 ± 141.21	<0.001
Total sugars (gm)	73.39 ± 54.95	101.42 ± 63.15	142.58 ± 89.96	<0.001
Total fat (gm)	56.91 ± 29.20	85.39 ± 36.99	118.19 ± 58.28	<0.001
Cholesterol (mg)	213.01 ± 192.92	311.74 ± 222.01	424.86 ± 302.00	<0.001
Calcium (mg)	574.15 ± 337.20	869.45 ± 403.83	1254.05 ± 669.72	<0.001
Caffeine (mg)	95.28 ± 114.44	136.05 ± 151.53	193.68 ± 291.78	<0.001
SBP (mmHg)	125.09 ± 20.46	123.89 ± 18.64	124.57 ± 17.81	0.265
DBP (mmHg)	75.61 ± 11.93	74.44 ± 11.26	74.31 ± 11.12	<0.001
BMI (kg/m2)	30.81 ± 8.01	30.18 ± 7.46	29.36 ± 7.01	<0.001
Glycohemoglobin (%)	5.89 ± 1.19	5.86 ± 1.12	5.83 ± 1.06	0.678
Fasting glucose (mmol/L)	6.21 ± 2.00	6.34 ± 2.26	6.33 ± 2.05	0.002
Creatinine, refrigerated serum (umol/L)	79.42 ± 42.33	78.15 ± 37.49	80.22 ± 34.84	<0.001
Globulin (g/L)	31.43 ± 4.38	30.97 ± 4.49	30.43 ± 4.17	<0.001
Cholesterol, refrigerated serum (mmol/L)	4.80 ± 1.06	4.81 ± 1.05	4.84 ± 1.07	0.295
Triglycerides (mmol/L)	1.50 ± 1.17	1.55 ± 0.98	1.67 ± 1.47	<0.001
Uric acid (umol/L)	318.73 ± 89.15	318.80 ± 87.20	326.21 ± 86.18	0.001
Gender (%)				<0.001
Male	37.26	45.37	63.42	
Female	62.74	54.63	36.58	
Race (%)				<0.001
Mexican American	9.50	11.44	13.95	
Other Hispanic	9.98	9.74	10.98	
Non-Hispanic White	31.37	38.08	37.77	
Non-Hispanic Black	34.83	24.98	20.37	
Other race	14.31	15.76	16.92	
Education level (%)				<0.001
Less than 9th grade	6.96	6.25	7.41	
9-11th grade	12.13	9.78	10.34	
High school graduate/GED or equivalent	29.38	22.84	20.13	
Some college or AA degree	33.64	34.60	31.83	
College graduate or above	17.89	26.53	30.29	
Marital status (%)				<0.001
Married/living with partner	51.41	60.06	62.47	
Widowed/divorced/separated	25.09	21.77	20.41	
Never married	23.5	18.17	17.12	
Drinking status (%)				<0.001
Yes	13.36	13.57	19.42	
No	86.64	86.43	80.58	
Hypertensive (%)				0.001
Yes	40.91	39.07	35.24	
No	59.09	60.93	64.76	
Pain or discomfort in chest (%)				0.479
Yes	31.33	30.47	29.38	
No	68.67	69.53	70.61	
Diabetes (%)				0.066
Yes	16.42	15.56	13.75	
No	83.58	84.44	86.25	
Vigorous work activity (%)				0.003
Yes	24.06	25.22	28.42	
No	75.94	74.78	71.58	
Smoke status				0.026
Yes	42.54	40.22	44.43	
No	57.46	59.78	55.57	
Albuminuria (%)				0.002
Yes	14.99	14.13	11.65	
No	85.01	85.87	88.35	

The mean ± SD of continuous variables: *p*-value is calculated using a weighted linear regression model categorical variable: *p*-value is calculated by weighted chi-square test.

**Table 2:** Association between dietary potassium intake and albuminuria.

	OR^a^ (95% CI^b^), *p*-value		
	Crude model (model 1)^c^	Minimally adjusted model (model 2)^d^	Fully adjusted model (model 3)^e^
Continuous	0.99 (0.98, 0.99) <0.0001	0.99 (0.98, 0.99) <0.0001	0.99 (0.98, 1.00) 0.0005
Categories			
Tertile 1	Reference	Reference	Reference
Tertile 2	0.93 (0.80, 1.09), 0.3884	0.92 (0.78, 1.08), 0.3153	0.91 (0.76, 1.09), 0.2967
Tertile 3	0.75 (0.64, 0.88), 0.0005	0.73 (0.62, 0.87), 0.0004	0.74 (0.61, 0.89), 0.0016
*p* for trend	0.0004	0.0004	0.0014

In the sensitivity analysis, dietary potassium intake is converted from a continuous variable to a categorical variable (quantile), where we set the third place.

a OR: odds ratio.

b 95%CI: 95% confidence interval.

c Model 1: no covariates were adjusted.

d Model 2: adjusted for gender, age, and race.

e Model 3: adjusted for gender, age, race, income of poverty ratio, energy, protein, carbohydrate, total sugars, total fat, cholesterol, calcium, caffeine, SBP, DBP, BMI, triglyceride, total Cholesterol, glycohemoglobin, fasting Glucose, creatinine, refrigerated serum, cholesterol, refrigerated serum, triglycerides, uric acid, education level, marital status, drinking status, hypertensive, pain, diabetes, vigorous work activity, smoke status, and albuminuria. Because the results were not obvious, we amplified dietary potassium intake by 100 times to study.

### 3.2 Relationship between albuminuria and dietary potassium intake

According to our research, a higher dietary potassium intake was linked to a lower risk of albuminuria. In our original model (*p* < 0.0001) and minimally adjusted model (*p* < 0.0001), there was a strong correlation between the two. This negative association between potassium intake and albuminuria remained significant in a fully adjusted model (*p* = 0.0005), indicating that each unit increase in potassium intake was associated with a 1% reduction in the risk of developing albuminuria. After conducting sensitivity analysis, it was discovered that patients in the third quartile had a statistically significant 26% lower incidence of albuminuria than those in the first quartile (*p* = 0.0016).

Table 3 is the multivariate logistic regression model for albuminuria. In fully adjusted models, age, education, smoking level, exercise level, BMI, SBP, DBP, hypertension, diabetes, uric acid, Triglycerides, total dietary protein intake, serum creatinine, and cholesterol were significantly (*p* < 0.05) associated with albuminuria. Subjects with 9-11th grade, High school graduate/GED or similar. Some college or AA degree, College graduate or above, and Subjects with Less Than 9th Grade Education were 35.3, 45.2, 48.4, and 63.2% Less Likely to Have Albuminuria, respectively. When compared to participants who engaged in vigorous activity regularly, those who did not had a 33.3% higher risk of developing albuminuria. Nonsmokers were 18.7% less likely to have albuminuria than smokers. At the same time, the possibility of non-hypertensive and non-diabetic albuminuria was reduced by 69.3 and 80.7%. For each unit increase of SBP and DBP, the possibility of albuminuria increased by 3.1 and 1.8%.

**Table 3:** Multivariate logistic regression model of albuminuria.

Variables	OR^a^ (95%CI^b^)	*p*-value
Dietary potassium intake	0.988 (0.983, 0.994)	0.00004
Age (year)	1.035 (1.031, 1.040)	<0.00001
Female (versus male)	0.950 (0.833, 1.083)	0.44197
Race (versus Mexican American)		
Other Hispanic	0.773 (0.579, 1.032)	0.08063
Non-Hispanic White	0.865 (0.695, 1.076)	0.19339
Non-Hispanic Black	1.068 (0.855, 1.335)	0.56034
Other race	0.864 (0.671, 1.114)	0.25960
Education level (versus less than 9th grade)		
9-11th grade	0.647 (0.491, 0.854)	0.00210
High school graduate/GED or equivalent	0.548 (0.429, 0.699)	<0.00001
Some college or AA degree	0.516 (0.408, 0.652)	<0.00001
College graduate or above	0.368 (0.286, 0.475)	<0.00001
Smoke status (no versus yes)	0.813 (0.712, 0.928)	0.00210
Vigorous work activity (no versus yes)	1.333 (1.137, 1.562)	0.00039
BMI (kg/m2)	1.022 (1.013, 1.030)	<0.00001
SBP (mmHg)	1.031 (1.028, 1.035)	<0.00001
DBP (mmHg)	1.018 (1.012, 1.024)	<0.00001
Hypertensive (no versus yes)	0.307 (0.268, 0.352)	<0.00001
Diabetes (no versus yes)	0.193 (0.167, 0.224)	<0.00001
Uric acid (umol/L)	1.002 (1.002, 1.003)	<0.00001
Triglycerides (mmol/L)	1.140 (1.087, 1.195)	<0.00001
Total Protein (g/L)	1.035 (1.019, 1.051)	<0.00001
Creatinine (umol/L)	1.016 (1.013, 1.018)	<0.00001
Cholesterol (mmol/L)	0.885 (0.828, 0.945)	0.00030

Total protein (g/L) is the amount of total protein in the serum. The unit of the continuous variable and the reference value of the non-continuous variable are moved next to the variable. In the continuous variable, the OR of proteinuria increased per unit, compared to the reference value in the categorical variable.

a OR: odds ratio.

b 95%CI: 95% confidence interval.

### 3.3 Subgroup analysis

**Figure 2:**
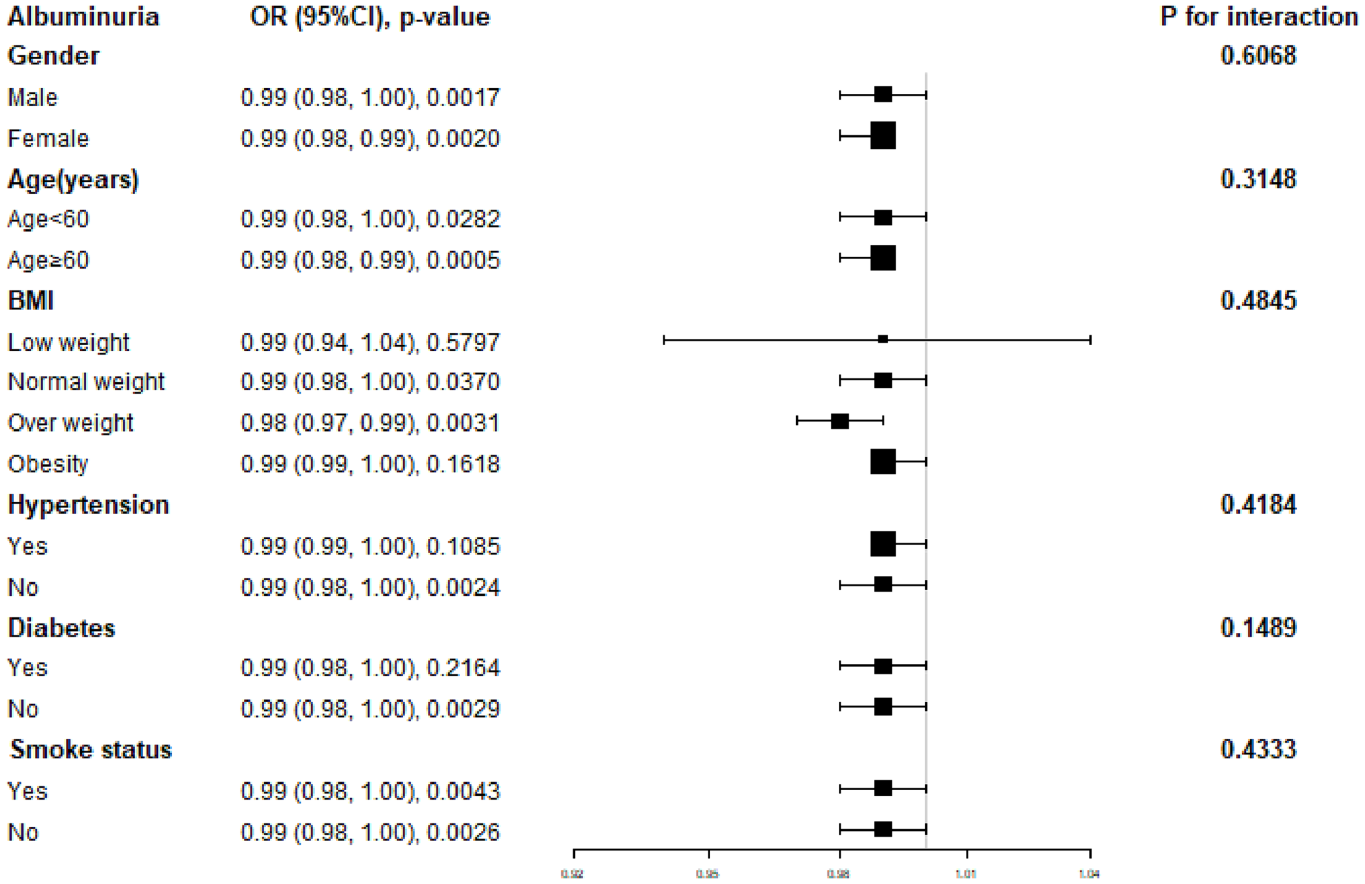
Subgroup analysis of association between dietary potassium intake and albuminuria.

Figure 2 displays a subgroup analysis of the relationship between dietary potassium intake and albuminuria. We discovered erratic relationships between dietary potassium intake and a potential decline in albuminuria prevalence. By sex, age, and smoking, potassium intake was significantly associated with albuminuria in each subgroup. Among BMI subgroups, the association of potassium intake with albuminuria was significant for normal weight and overweight subgroups, but not statistically significant for lower weight and obese subgroups. Simultaneous interaction tests indicated that gender, age, BMI, hypertension, diabetes, and smoking had no significant dependence on the negative association between potassium intake and albuminuria.

## 4 Discussion

Based on the inclusion and exclusion criteria, 7,564 people participated in the study throughout the National Health and Nutrition Examination Survey (NHANES) cycle from 2017 to 2020. We discovered that people who consumed more potassium through their diets had a lower likelihood of having albuminuria. Interaction tests and subgroup analysis suggest that this link may hold across populations. Our findings suggest that consuming a diet rich in potassium may raise the likelihood of developing albuminuria.

Potassium is one of the important minerals for maintaining normal physiological functions of the human body and is also very important for kidney health. Proteinuria refers to the amount of protein in the urine that exceeds the normal range and is often an important indicator of kidney disease. Current studies have been inconsistent on the effect of potassium intake on the likelihood of proteinuria. Some studies have found that moderate potassium intake can reduce the risk of proteinuria because potassium can help maintain normal blood pressure and kidney function. For example, a study published in the Journal of the American College of Cardiology found that people who consumed 2,000 to 4,000 milligrams of potassium per day had a 30 percent lower risk of proteinuria compared to those who consumed less than 2,000 milligrams of potassium per day. However, some studies have found that excessive potassium intake may increase the risk of proteinuria. For example, a study published in the American Journal of Clinical Nutrition found that people who consumed more than 5,000 mg of potassium per day had a 40 percent increased risk of proteinuria compared to those who consumed less than 2,000 mg of potassium per day. Overall, moderate potassium intake is beneficial for maintaining kidney health and reducing the risk of proteinuria. However, excessive potassium intake may increase the risk of proteinuria. Therefore, it is recommended that people according to their health conditions and dietary habits, moderate intake of potassium. If you have kidney disease or other health problems, you should adjust your potassium intake under the guidance of your doctor.

According to certain research, inadequate dietary potassium consumption may cause a decline in renal function, which raises the risk of albuminuria. This is about the impact of dietary potassium intake on renal function (26). According to Wu et al., Kir5.1, which is encoded by Kcnj16, may modulate the connection between dietary potassium intake and renal potassium excretion (27). In this study, the authors demonstrate that deleting Kir5.1 eliminates the inhibitory effect of high dietary potassium intake on NCC and impairs the kidney’s ability to excrete potassium when dietary potassium intake is increased. Their findings suggest that Kir5.1, like Kir4.1, is also an important component of the potassium sensing mechanism in the distal convoluted tubules, and that Kir5.1 is essential for regulating renal potassium excretion and maintaining potassium homeostasis. Claudino et al. (28) concluded that chronic kidney disease allows for small potassium intakes. The presence of residual renal function is an important factor affecting potassium excretion, and a low-restriction diet can be adopted. Potassium can affect blood pressure in addition to renal function. Increased potassium intake, according to Lawrence J. Appel, may lower blood pressure (29). High potassium intake is good for the human body, including hypertension, urolithiasis, and osteoporosis, according to research by Penton et al. on the relationship between dietary potassium intake and renal sodium and potassium processing (30). Dietary potassium is protective (31). Therefore, in the selection of covariates, we chose blood pressure. Beyond that, potassium intake may be linked to cognitive performance. Nowak et al. found that higher potassium intake may lead to cognitive decline in the elderly (24). In a Korean cohort study conducted by Mun et al., glomerular filtration rate was linked to dietary potassium and a lower risk of developing chronic kidney disease. Adequate potassium intake has also been shown to be beneficial in the treatment of hypertension (32). Blanch et al. (33) found that increasing potassium intake was effective in improving vascular function. Many studies have suggested that high potassium intake is beneficial, however, Virojanawat et al. (34) found that low potassium intake may be the key factor for hypokalemia in chronic peritoneal dialysis patients in Thailand, rather than potassium conversion in cells or potassium excretion. Due to the kidney’s crucial role in the body’s potassium homeostasis, low potassium levels will cause the glomerular filtration rate to drop, which will influence the excretion of albumin. Like in most research, albuminuria can be decreased by minor increases in dietary potassium intake.

Effect of albuminuria on dietary potassium intake, Kabasawa et al. found a positive correlation between dietary sodium-potassium ratio and albuminuria (14) but did not study the relationship between potassium and albuminuria. This study found that potassium may be a key factor affecting albuminuria. Our study is meaningful. There is no study investigating the relationship between the two from NHANES. Kabasawa et al. (14) discovered a potential inverse link between potassium intake and albuminuria in another investigation. The research results of Burnier et al. (35) and Elfassy et al. (36) are opposite, and our research results are consistent with Burnier et al., increasing potassium intake may reduce the incidence of albuminuria. Influence of Other Factors on Dietary Potassium Intake and Albuminuria in addition to renal function and albuminuria, many other factors may affect dietary potassium intake and the incidence of albuminuria. For example, factors such as age, sex, body mass index (37), diabetes (38), etc. may have an impact on these indicators. In addition, different cultural backgrounds and dietary habits can also affect dietary potassium intake. As a result, while analyzing the connection between dietary potassium intake and albuminuria, the combined impact of these factors must be taken into account. The development of this study is crucial because it has a wide range of clinical application value and market potential. Because previous studies on the association between potassium intake and albuminuria were inconsistent, it was difficult to draw firm conclusions.

No mechanism is fully understood to explain the link between dietary potassium intake and albuminuria. However, some studies have shown that this link may be related to the following aspects: the regulation of potassium by the kidney (39), an important organ for maintaining potassium homeostasis in the body, which can regulate the concentration of potassium in the blood through mechanisms such as glomerular filtration, renal tubular secretion, and reabsorption. Changes in cell membrane permeability (40). Albumin is an important plasma protein whose presence can affect cell membrane permeability. Several studies have suggested (41) that cell membrane permeability may be altered in patients with albuminuria, resulting in reduced renal potassium reabsorption. Therefore, an appropriate increase in dietary potassium intake can help improve cell membrane permeability, thereby reducing the incidence of albuminuria.

The mechanism of the influence of dietary potassium on renal function and proteinuria mainly has the following aspects: Maintain normal blood pressure: Potassium can help vasodilate and lower blood pressure, thereby reducing the burden on the kidneys and reducing the risk of proteinuria. Promote sodium excretion: Potassium can promote the excretion of sodium by the kidneys, thus reducing the amount of sodium in the body and lowering the risk of blood pressure and proteinuria. Tubule protection: Potassium protects tubule epithelium and reduces tubule damage and inflammation, thereby reducing the risk of proteinuria. Regulating acid–base balance: Potassium can help maintain acid–base balance in the body, which reduces the burden on the kidneys and reduces the risk of proteinuria.

This study has the advantage of using trustworthy NHANES data and a sizable sample size. Second, we looked into previous research and included all necessary factors in this study. Because this study is cross-sectional, there is no causal association between the variables, which is a drawback. The effects of dietary potassium on renal function and proteinuria have certain limitations, as follows: The effects of potassium intake vary from person to person: different people have different kidney function and metabolism, and different dietary potassium requirements. Therefore, for some people, consuming too much dietary potassium may be a burden on the kidneys and increase the risk of proteinuria. Sources and absorption of dietary potassium: Sources of dietary potassium include fruits, vegetables, nuts, legumes and other foods, some of which also contain other beneficial nutrients. In addition, the absorption of dietary potassium is also affected by intestinal flora. Therefore, simply increasing dietary potassium intake is not necessarily effective in improving kidney function and reducing the risk of proteinuria. Influence of other factors: The occurrence and development of renal function and proteinuria are affected by many factors, including hypertension, diabetes, obesity and so on. Therefore, relying solely on dietary potassium to improve kidney function and reduce the risk of proteinuria may not be effective enough, and treatment considering other factors is also needed. The causal relationship between factors must therefore be explained by numerous prospective studies.

## 5 Conclusion

Reduced risk of albuminuria was linked to higher dietary potassium intake. The relationship between the two still needs further prospective investigations, though.

## Data availability statement

The original contributions presented in the study are included in the article/supplementary material, further inquiries can be directed to the corresponding author.

## Ethics statement

The studies involving humans were approved by the National Center for Health Statistics Institutional Review Board. The studies were conducted in accordance with the local legislation and institutional requirements. The participants provided their written informed consent to participate in this study.

## Author contributions

WYua: Conceptualization, Data curation, Formal analysis, Funding acquisition, Investigation, Methodology, Visualization, Writing – original draft, Writing – review & editing. TW: Conceptualization, Data curation, Formal analysis, Funding acquisition, Resources, Software, Visualization, Writing – original draft, Writing – review & editing. WYue: Conceptualization, Funding acquisition, Investigation, Methodology, Project administration, Resources, Supervision, Validation, Writing – original draft, Writing – review & editing.
